# Supplementary material for: Deletion of wfs1 Impairs Oligodendrocyte Precursor Cells Dorsal Distribution and Myelination Through the wfs1-hmgcs1 Axis in Zebrafish
Source: Biology (Basel). 2026 Aug 16;15(16):1402. doi: 10.3390/biology15161402 (PMC13510151; doi:10.3390/biology15161402)
Supplement: Supplementary file 1 [file biology-15-01402-s001.zip › File S1-Transcriptome sequencing data and differentially expressed genes (DEGs) in wfs1-- zebrafish at 5 dpf/2.KEGG/wfs1KOvsWT/UP/src/dre04141.html]

dre04141


- dre:556410

- Up regulated genes

556410\_1(1.7264669285964)

- dre:327197

- Up regulated genes

327197\_1(2.29609005360308)

- dre:567021

- Up regulated genes

567021(7.12614903992843)

- dre:566876

- Up regulated genes

566876(1.88165913028669)

- dre:573376

- Up regulated genes

573376(2.09344333483373)

- dre:574002

- Up regulated genes

574002(4.26297879374466)

- dre:573376

- Up regulated genes

573376(2.09344333483373)

- dre:386590

- Up regulated genes

386590\_1(9.5728659061572)

- dre:266757

- Up regulated genes

266757(2.51574060189836)

- dre:567029

- Up regulated genes

567029\_1(1.92851492715724) 108181182(4.01339782525625)

- dre:337190

- Up regulated genes

337190(2.47608711107746)

Close
